# Genetic and Association Mapping Study of Wheat Agronomic Traits Under Contrasting Water Regimes

**DOI:** 10.3390/ijms13056167

**Published:** 2012-05-18

**Authors:** Dejan Dodig, Miroslav Zoric, Borislav Kobiljski, Jasna Savic, Vesna Kandic, Steve Quarrie, Jeremy Barnes

**Affiliations:** 1 Maize Research Institute, Slobodana Bajica 1, Belgrade-Zemun Polje 11185, Serbia; E-Mail: vkandic@mrizp.rs; 2 Environmental and Molecular Plant Physiology Research Group, School of Biology, Devonshire Building, Newcastle University, Newcastle upon Tyne NE1 7RU, UK; E-Mails: steve.quarrie@googlemail.com (S.Q.); jerry.barnes@ncl.ac.uk (J.B.); 3 Institute of Field and Vegetative Crops, Maksima Gorkog 30, Novi Sad 21000, Serbia; E-Mails: miroslav.zoric@ifvcns.ac.rs (M.Z.); borislav.kobiljski@ifvcns.ac.rs (B.K.); 4 Faculty of Agriculture, University of Belgrade, Nemanjina 6, Belgrade 11080, Serbia; E-Mail: jaca@agrif.bc.ac.rs

**Keywords:** wheat, agronomic traits, drought, population structure, association mapping

## Abstract

Genetic analyses and association mapping were performed on a winter wheat core collection of 96 accessions sampled from a variety of geographic origins. Twenty-four agronomic traits were evaluated over 3 years under fully irrigated, rainfed and drought treatments. Grain yield was the most sensitive trait to water deficit and was highly correlated with above-ground biomass per plant and number of kernels per m^2^. The germplasm was structured into four subpopulations. The association of 46 SSR loci distributed throughout the wheat genome with yield and agronomic traits was analyzed using a general linear model, where subpopulation information was used to control false-positive or spurious marker-trait associations (MTAs). A total of 26, 21 and 29 significant (*P* < 0.001) MTAs were identified in irrigated, rainfed and drought treatments, respectively. The marker effects ranged from 14.0 to 50.8%. Combined across all treatments, 34 significant (*P* < 0.001) MTAs were identified with nine markers, and *R*^2^ ranged from 14.5 to 50.2%. Marker psp3200 (6DS) and particularly gwm484 (2DS) were associated with many significant MTAs in each treatment and explained the greatest proportion of phenotypic variation. Although we were not able to recognize any marker related to grain yield under drought stress, a number of MTAs associated with developmental and agronomic traits highly correlated with grain yield under drought were identified.

## 1. Introduction

Environmental stresses constitute the main factor depressing wheat production across the world, with wheat yield particularly suppressed by untimely temperature extremes and water deficit. This situation is emphasized by the 25 and 18% reduction in durum yields experienced across Southern Europe in 2003 and 2005, respectively, attributable to the high temperatures and water shortages experienced by crops in the field in this region in these years, compared with 2004 [1]. Depending on the timing, duration and intensity of temperature extremes/drought, wheat yield maybe depressed by 10% to 90% [2].

Climate change models project that summer rainfall will decrease substantially (in some areas up to 70%) across Southern and Central Europe [3] and many—like that of Döll and Flörke [4]—predict that summer droughts experienced during grain filling like those experienced in 2003 and 2005 will constitute a growing influence limiting European wheat production, especially at Southern latitudes and in parts of Eastern Europe. Temperatures during grain-filling already regularly exceed 30 °C, and the Intergovernmental Panel on Climate Change [5] predicts the Mediterranean region as a whole will be likely adversely affected. Given current pressures on food security it is vital that breeders strive to maintain wheat yields by improving crop adaptability, stability and sustainability in a bid to combat a multitude of environmental challenges, not least water shortage. In this context, a better understanding of the genetic control of yield and the main traits that underlie the adaptive response of crops across a broad range of water availabilities is essential for more effective and targeted breeding activity [6].

The development of molecular marker technologies to identify a particular chromosomal location of genes regulating specific traits has revolutionized our understanding of the genetic control of these traits. Quantitative trait loci (QTL) analysis and more recently association mapping (AM), exploiting linkage disequilibrium [7], have become valuable tools in identifying the genetic control of yield and agronomically-relevant traits across a range of environments [8–11]. By performance comparison of the same genotypes across environments it is possible to identify those environments in which particular QTLs are expressed. Association analysis based on elite lines and breeding material provides a particularly useful tool to detect loci for traits with low heritability such as yield and its components [12,13]. However, other traits associated with wheat adaptability to soil water deficit (e.g., phenological and physiological traits, plant height, harvest index), with higher heritability than grain yield, can be also employed to improve yield in drought-stressed environments [10,14–17].

The choice of germplasm is a key factor determining the resolution of AM. In order to detect more alleles, the germplasm selected should theoretically include all the genetic variation of a specific species because diverse germplasm includes more extensive recombination during its history and allows a high level of resolution [18]. A species for which a core collection has been established would constitute the ideal material for AM [19]. In this study we used a core collection of 96 winter wheat accessions sampled from 21 countries across five continents as the AM population. The objectives of this research were: (a) to undertake genetic analysis of the AM population; and (b) to identify chromosome regions that are highly correlated with yield and agronomic traits under contrasting environments (near optimal *versus* rainfed and moderate-to-severe soil water deficit).

## 2. Results

### 2.1. Phenotypic Evaluation

The analysis of variance for each treatment revealed highly significant differences (*P* < 0.01) in all 24 studied traits between the 96 genotypes (data not shown). The mean value across three years in each treatment is presented in Table 1. Significant differences (*P* < 0.05) between irrigated (IP) and drought (DP) were found for all traits except stem height (SH), peduncle length (PL), peduncle extrusion (PE), spike density (SD), spike index (SI) and flag leaf chlorophyll content at flowering date (CH1). The average reduction due to drought (sheltered plots) ranged from 1.4% for SI to 64.5% for grain yield (GY) (Table 1). GY along with above-ground biomass per plant (BPP) (62.3% reduction) and kernel number per square meter (KN) (60.3%) were the traits most affected by drought stress. In addition, drought stress resulted in a significant increase in sterile spikelets per spike (SSS) (62%), and caused heading and flowering to be accelerated by 4–5 days across all genotypes. In most cases the values of the traits in the rainfed plots (RP) were intermediate between those of the IP and DP. The estimated variance of components of random effects which demonstrate the importance of accessions, environment and their interactions on each of 24 agronomic traits is presented in Electronic Supplementary Table 1.

### 2.2. Heritability of the Traits

The estimates of overall heritability (combined across all treatments) and within each treatment are presented in Table 2.

The overall heritability was high (>0.75) for days to heading (DTH), days to flowering (DTF), SH, PL, PE, spike length (SL), SD, fertile spikelets per spike (FSS), SSS, kernels per spike (KS), 1000 kernel weight (TKW), harvest index (HI), production per spike (PPS), flag leaf area (LA) and flag leaf width (LW), moderately high (0.50–0.75) for kernels per spikelets (KSL), SI, CH1, flag leaf chlorophyll content at grain filling (CH2) and GY and low for KN (0.48), BPP (0.44) and days between heading and flowering (HF) (0.37). The heritability in IP ranged from 0.46 (HF) to 0.93 (SL) and in DP from 0.26 (HF) to 0.89 (SD). In general, heritability values were slightly lower under drought than irrigated condition. However, the heritability estimate for GY under drought stress was rather lower than the same value obtained for irrigated plots (0.47 *vs.* 0.74, respectively). This is also true for BPP (0.39 *vs*. 0.64) and KN (0.38 *vs*. 0.61). Among main yield components (PT, KS and TKW) the highest heritability in DP was KS (0.78) while in IP was TKW (0.87).

### 2.3. Genetic Correlations

The genetic correlations between GY and other studied traits, including drought susceptibility index (DSI) and stress tolerance index (TOL), across all treatments and within each treatment are presented in Table 3.

Across all treatments significant positive correlations ranged from 0.21 (KS) to 0.94 (TOL) and significant negative correlations ranged from −0.22 (HF) to −0.42 (SSS). In plats subject to drought, GY was highly associated with BPP (*r*_g_ = 0.86), KN (*r*_g_ = 0.81) and TOL (*r*_g_ = 0.72). Higher grain yield in DP was moderately associated (0.30 < |*r*_g_| < 0.58) with KS, SH, CH2, PT, FSS, CH1, HI, PPS and TKW and, to lesser extent with lower SSS (*r*_g_ = −0.30) and shorter DTH (*r*_g_ = −0.28), DTF (*r*_g_ = −0.28) and HF (*r*_g_ = −0.25). Several traits such as FSS, KS and LA appeared to be significant for GY in DP but not in RP and/or IP. In contrast, DTH, DTF, PL and PE were significant for GY in RP and/or IP treatments, but not under drought. In IP and RP treatment the highest negative correlation was with SSS (*r*_g_ = −0.42 and −0.41, respectively) while the highest positive correlation was with TOL (*r*_g_ = 0.97 and 0.95, respectively). Note that for TOL positive correlations it should be interpreted as negative, because a higher TOL value indicates greater susceptibility, not a higher level of tolerance.

### 2.4. Model-Based Groups within AM Population and Linkage Disequilibrium

The genetic relationships among the accessions were investigated using a model-based Bayesian clustering method. A higher number (>4) of Bayesian clustering-based subgroups improved the overall fit to the model but led to a general decrease in the number of accessions assigned to a specific subgroup with high membership probability (>0.80), suggesting excessive partitioning of the diversity structure. The accessions included in each of the four subgroups (A–D) together with their corresponding membership probability estimates are reported in Electronic Supplementary Table 2.

The effect of population structure on phenotypic traits was investigated by means of regression analysis (Table 4).

Using the mean values across treatments, the greatest effect was observed for stem related traits SH, PL and PE (*R*^2^ = 57.8%, 53.3 % and 42.9%, respectively); a modest influence of population structure was detected for sterile spikelets per spike (SSS), CH1, CH2, HI and BPP, with *R*^2^ values ranging from 12.6% to 25.6%. Interestingly, the effect of population structure on GY and its components (PT, KN, TKW), including earliness (DTH and DTH), was low (*R*^2^ ranged from 1.2% to 6.4%). Similar results were also obtained when considering data of each treatment (data not presented).

**Figure 1:**
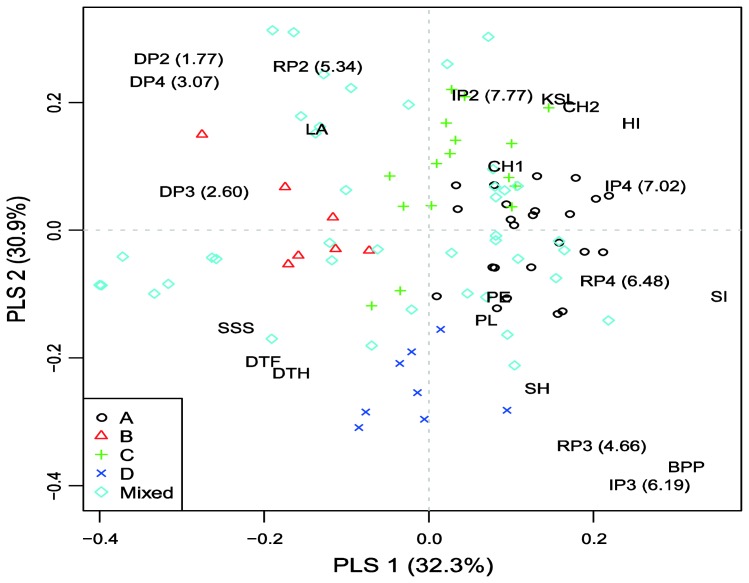
Triplot of the first and second partial least squares (PLS) components for agronomic traits for 96 wheat accessions tested across 9 environments. Environment codes are: IP, irrigated plot; RP, rainfed plot; DP, drought plot, 2, 2002 year, 3, 2003 year; 4, 2004 year. Number in parentheses (*x*) represent average yield across all accessions. Accessions are denoted according to subpopulations assignments and indicated by different symbols on the figure. Trait codes: BPP = above-ground biomass per plant; CH1 = flag leaf chlorophyll content at flowering date; CH2 = flag leaf chlorophyll content three weeks after flowering date; DTF = days to flowering; DTH = days to heading; HI = harvest index; KSL = number of kernels per spikelet; LA = flag leaf area; PE = peduncle extrusion; PL = peduncle length; SH = stem height; SI = spike index; SSS = sterile spikelets per spike.

To explore model-based group × environment (treatment/year combination) interactions a partial least squares (PLS) regression approach was adopted using agronomic traits as explanatory variables. Only traits significantly affected (*P* < 0.05) by population structure were included in the analysis (see Table 4). The results of PLS regression for grain yield (denoted according to sub-populations assignments, environments and agronomic traits affecting interactions across the 96 accessions) is presented in Figure 1.

Most accessions from sub-populations A and C are characterized by high chlorophyll content, KSL and HI, as well as low DSI and shorter vegetation, and had positive interaction with more favorable environments (IP2 and IP4). Accessions from sub-population B had low stature and large leaf area and showed positive interactions with drought stress environments. Accessions from sub-population D were late and tall and showed positive interactions in medium yielding environments (RP3 and IP3). Generally, the results from PLS regression for exploring specific interactions for grain yield in the trial indicated similar responses of accessions within model-based groups to environmental factors.

The extent of genome-wide LD among the entire set of accessions was evaluated through pairwise comparisons among 46 SSR loci yielding 1035 estimates. Among them, 178 (17.2%) showed significant association at a comparison-wise 0.01 level. The pairwise *r*^2^ estimates among 46 SSR loci ranged from 0 to 0.606, with a mean of 0.020 and a median of 0.015 (data not shown).

### 2.5. Marker-Trait Association

Using subpopulation assignments as covariates, a total of 517 significant marker–trait associations (MTAs) for 24 agronomic traits were detected in IP, RP and DP treatments at 41 loci. Fifty-eight percent of the associations were significant at the 0.05 level, 30% at the level 0.01 and 12% at the level 0.001. In the following special emphasis is given to highly significant results (with *P* < 0.001); findings with lower significance are not reported. A total of 64 highly significant MTAs were identified in IP (24), RP (16) and DP (24) with 11 different SSR markers, and *R*^2^ ranged from 11.4 to 42.2% (Table 5). Thirty seven associations survived a Bonferroni correction for multiple testing (Table 5). Combined across all treatments 28 significant (*P* < 0.001) MTAs were identified with nine markers (*R*^2^ ranged from 13.4 to 41.4%) while 15 associations survived Bonferroni correction (Table 5).

DTF was involved in the highest number of MTAs within three treatments (9), followed by PT (7) and DTH, SL and FSS (all 6), while the fewest MTAs were associated with KN, GY and CH2 (all 2). No associated marker was found for HF, SH, PL, PE, SD, SI, TKW, BPP and CH1 within treatments. Of a total of 11 markers which showed highly significant effect on traits, eight (gwm99, gwm130, gwm369, gwm389, gwm458, gwm 540, psp3050 and psp3088) were associated with only one trait and therefore can be called trait-specific MTAs; the other three (gwm257, gwm484 and psp3200) were associated with more than one trait and can be referred as multi-trait MTAs. Marker gwm484 was found to be associated with 10 traits in three treatments and both drought indices (explained up to 42.2% of the phenotypic variation), while psp3200 was observed significant associated with nine traits in three treatments (explained up to 31.8% of the phenotypic variation). Marker gwm257 was associated with DTH, DTF and TOL (explained up to 16.3% of the phenotypic variation).

## 3. Discussion

We report a genetic analysis and a SSR-based association study of a breeder’s core collection of 96 winter wheat accessions of diverse origin, evaluated for a set of 24 agronomic traits and two drought indices over three years and three treatments (nine environments *in toto*). The 96 accessions were chosen from a wider collection of wheat genotypes from the, Novi Sad, Serbia with the aim to accumulate as much variation as possible within traits of importance for wheat breeding programs. Treatments allowed us to measure yield and other traits across a range of water availabilities; from 54.3 mm (drought plots in 2002–2003 season) to 418.2 mm (irrigated plots in 2003–2004 season). A rain-out shelter was effective in providing a drought treatment in each season; average yield reductions in comparison with the irrigated treatments were 77.2%, 58.0%, and 56.3% in seasons one to three, respectively.

Considerable phenotypic variation in GY [coefficient of variation (CV) = 24.8%)] and other measured traits [CV ranged from 6.2% (CH1) to 82.2 (SSS)] was observed across the 96 accessions. The total number of alleles per SSR locus (7.96) and estimated gene diversity (0.64) previously reported for this core collection [20,21] are comparable with that reported in the wheat elite germplasm collections that have been molecularly characterized so far [11,22–24] and confirm the broad genetic base of the 96 accessions comprising the core collection investigated. Higher heritability estimates for GY under IP and RP than DP supported previous evidence that significant genotype by environment interactions exist under drought conditions [10,25,26]. Low heritability for BPP and more predictable genotype by environment interaction and little effect of the environment on SH, HI and PPS were also suggested in earlier studies [10,27,28].

The percentage of SSR loci pairs in LD observed in our mapping population (17%) was comparable with reports in maize (10%) [29] and cotton (11–12%) [30]. However, results from a SSR survey of other small grain cereals, including cultivated barley [31], elite durum wheat germplasm [32] and bread wheat collection [22], showed much high levels of LD (45–82%). This difference could be due, in part, to the small number of used loci (46) in this study which may give limited genome coverage with only 2–3 markers per chromosome. The wide genetic diversity found among the 96 accessions [20,21] may have also contributed to lower levels of LD detected.

Among the defined sub-populations, the smallest sub-population (B) is the most easily distinguished (there were no overlapping points with other subgroups) (Figure 1). The most distinct from B is sub-population D. Phenotypically, D accessions were significantly taller (stem height = 107.7 *versus* 54.7 cm) and flowered seven days later than B accessions. Sub-population B was dominated by accessions from Western Europe (GBR and FRA) while D centered on accessions of USA origin (Electronic Supplementary Table 2). It might be surprising that relatively late and short genotypes from sub-population B showed positive interaction with drought conditions (Figure 1). According to the conceptual model for drought resistance [2], taller plants are considered to have better yield stability under adverse conditions, while shorter plants are better adapted to irrigated and high-input environments. Also, shortening of the growing season has been a very successful strategy when breeding wheat for variable rainfall conditions [33]. Possibly an appropriate balance between water use during plant development and water use during grain fill existed in the genotypes from sub-population B. Hence, this sub-population may be of great interest to explore further for drought tolerance. The least differentiated pair was A–C. Sub-population C was the most diverse among sub-populations (spread over three quadrants) and consisted of genotypes from five continents. Sub-population A (mainly consisted of European accessions) exhibited positive interaction with irrigation treatments. This is not unexpected since many wheat-breeding programs in Europe are being carried out under non-limiting conditions.

We used sub-population assignments as covariates in applied AM for identification of genetic markers associated with grain yield and agronomic traits. Many studies in the past have demonstrated that all seven chromosome groups are involved in the genetic control of yield and yield-related traits in bread wheat [34]. Due to the complex genetic control of grain yield, we found only two markers (gwm99 and gwm484) linked to genomic regions contributing to grain yield. Both were associated with GY only in well watered conditions. This is in keeping with Maccaferri *et al.* [11] who suggested that as the level of moisture stress increases, the power to detect the relevant loci for grain yield via AM decreases. They explained that under such conditions similar grain yield values can be attained by different genotypes through different adaptive strategies and corresponding gene networks, thus undermining the occurrence of significant marker–trait associations.

Markers gwm99 (on chromosome arm 1AL) and gwm484 (on chromosome arm 2DS) explained relatively high portion of the phenotypic variation of GY (14.4 and 22.3%, respectively). Furthermore, gwm484 effect for GY combined across all treatments was also rather high (19.8%). Such a high percentage of explained variations of GY by QTLs are not so common in association studies in cereals. Previous studies have also shown regions on chromosome arms 1AL and 2DS associated with grain yield in bread wheat. Using a meta-QTL analysis (MTQL), Zhang *et al.* [34] located three MTQLs for yield and yield-related traits on chromosome arm 1AL. One of them was positioned at 115 cM away from the terminal of 1AL which is ~11 cM from gwm99 based on the consensus map Ta-SSR-2004 [35]. In addition, Zhang *et al.* [34] identified four MQTLs for yield and yield-related traits on chromosome arm 2DS, located between 17 and 50 cM. Marker gwm484 is positioned within this interval (41 cM) based on the consensus map Ta-SSR-2004. A significant QTL for grain yield at 35 cM on chromosome arm 2DS was located by Kumar *et al.* [36]. In our study loci gwm99 was associated only with GY in only IP and this can be referred to as trait- and treatment-specific MTA. On the other hand loci gwm484 (2DS) was associated with a number of other agronomic and developmental traits including DTF, PT, SL, FSS, SSS, HI, LA, LW and CH2. Significant genetic correlations were observed between GY and most of these traits suggesting pleiotropy and/or linked loci controling them.

The highly-significant association between allele size and different traits, including DTF, at the locus gwm484 is probably due to the proximity to *Ppd-D1* photoperiod sensitivity gene on 2DS [37]. This gene is well known as having an influence on wheat yield through the optimization of flowering time [38]. Recently the existence of at least six haplotypes of *Ppd-D1* has been reported [39], with apparent adaptive significance, suggesting many more opportunities for fine-tuning genotypes to environmental conditions. It is typical that population structure coincides strongly with origin, adaptation and earliness, as was found in the present study. To minimize the effects that a high variability in phenology could have on GY under varying water regimes Maccaferri *et al.* [11] purposely kept the heading date within a narrow range when assembling a durum wheat mapping population. The correlation of earliness and GY was often significant suggesting that even relatively small range in heading date can significantly influence adaptation to the conditions commonly present in Mediterranean environments.

Besides gwm484 another microsatellite locus, psp3200 on chromosome arm 6DS, was associated with a large number of traits and involved in many MTAs, explained from 12.7% to 33.0% of phenotypic variation. As in the case of gwm484 locus most of detected MTAs for locus psp3200 survived a Bonferroni correction for multiple testing. The genomic region on the chromosome arm 6DS had a considerable effect on PT, SL, FSS, KS, PPS, LA, LW under optimum and water deficit conditions. Although no major flowering time effect has yet been reported on the short arm of chromosome 6D of wheat, the marker psp3200 was found to have highly significant allele associations with DTH and DTF under non-optimum conditions. This possibly new source of heading/flowering time gene(s) on chromosome arm 6DS could provide new opportunities for breeders to adapt their varieties better to the variable rainfed conditions, and under drought conditions early heading/flowering is generally advantageous. Recently, McIntyre *et al.* [10] reported a QTL for increased grain yield on chromosome 6B which co-located with a possible QTL for increased water-soluble carbohydrates (WSC) concentration in stem. A high stem carbohydrate concentration is considered to be a potentially useful trait for improving grain weight and yield in water-limited wheat production environments [40,41]. High WSC lines appeared to flower earlier than low WSC lines [10,42].

Although breeding for GY is the ultimate way to produce stress tolerant crop plants, due to the low heritability and complexity of grain yield, other traits such as yield components can be employed. Grain yield can be divided into a number of components including PT, KS and TKW. Several genes also control yield components; however, these components are usually less environmentally sensitive and have higher heritability than grain yield [43]. This is confirmed in our study as PT, KS and TKW had higher heritability than GY in all three treatments as well as across all treatments. However there were no significant MTAs for TKW, while three and two markers were detected to be associated with PT and KS, respectively. Markers gwm484 (2DS) and psp3200 (6DS) were associated with PT in more than one treatment and both were related to several other traits. Markers gwm389 (3BS) was trait-specificand related to PT only in DP, explaining 14.4% of the phenotypic variation. The existence of QTLs for the tiller number was also observed on chromosomes 1A, 1B, 2B, 4B, 5B and 6A [10,28].

For KS one trait-specific and one multi-trait MTAs were located on 5BS (gwm540) and 6DS (psp3200), respectively. Both MTAs were detected under non-stress conditions and across all treatments. In the study by Quarrie *et al.* [8] the yield component most strongly associated with yield QTLs clusters was KS. Other studies also indicate a high correlation between GY and KS [44,45]. In our study KN (which combines PT and KS) was the trait most highly associated with GY across all treatments. Marker related to increased KN was detected on chromosome arm 2AL (psp3088) for IP and DP treatments as well as across all treatments. In a recent study, QTLs for increased KN were found on chromosomes 1A and 7A both in bread [10] and durum wheat [11].

In addition to gwm99 another two trait- and treatment-specific markers were identified on chromosomes 1DL and 7AS. Marker gwm458 (1DL) was related to HI while marker gwm130 was associated with PPS, both in DP, explaining 12.2% and 15.3% of the phenotypic variance, respectively. Both traits were highly correlated with GY in all treatments and can be effective in contributing GY in different conditions. Golobadi *et al.* [28] located MTA for HI detected only under drought stress on chromosome 2B, which explained 26.5% of the phenotypic variance, while the most significant marker for HI under normal conditions was located on 4B chromosome. Specific MTAs for HI in bread wheat were also reported by Neumann *et al.* [13] on 1A, 3A, 7A and 7B chromosomes. Besides the MTA related to PPS (gwm130), chromosome arm 7AS harbored MTA for LW (psp3200) also only under drought stress. The variation in flag leaf width was found to be related with major yield QTL on 7AL chromosome, expressed mainly under stressed conditions [46]. The differences in width of flag leaves associated with the yield QTL on 7AL were due to variation in numbers of cell files across the leaf, *i.e.*, variation in cell division during leaf ontogeny. In recent studies QTLs for PPS were reported on several chromosomes including 7A short arm [10,13,28].

Three MTAs were found for drought indices on chromosome arms 2BS (gwm257) and 2DS (gwm484), explaining a relatively high proportion of the observed phenotypic variation (14.9 to 25.3%) for drought tolerance. Not surprisingly, markers gwm257 and gwm484 were also associated with variation in DTH and/or DTF as they are known to map close to photoperiod sensitivity genes *Ppd-B1*and *Ppd-D1,* respectively [47]. Genomic region on chromosome arms 2DS also exerted a considerable influence on SL, FSS, SSS, HI, LA, LW, CH2 (under drought stress) and PT, SL, FSS, SSS, HI, LA and GY (under optimum conditions). This finding concurs with that of Kumar *et al.* [36] who explained that a large proportion of phenotypic variation was not only consistent over environments, but was also pleiotropic, and/or coincident with QTLs for other traits. The coincidence of the gwm484 locus on chromosome 2DS with a number of traits across treatments may explain its effect on adaptation to different environmental conditions. The reports of QTLs for water-soluble carbohydrates [14,17] osmotic potential, chlorophyll content and flag leaf rolling [48] in homoelogous Triticae group 2 chromosomes highlight the importance of this chromosome group for physiological responses to drought stress of wheat. In previous QTL mapping studies in biparental populations the QTLs controlling drought tolerance indices have been reported on chromosomes 5B for TOL [49] and 4A, 4B, 5B, 7A for DSI [44,49], explaining between 13 and 41% of phenotypic variation.

Many of the reported MTAs within treatments were observed also in combined analysis across treatments. However, as a result of marker × environment interactions, not all these MTA are expressed in all environments (treatment/year combination). Nevertheless, the (pleiotropic) effect on spike morphology (SL and FSS) appears to be expressed in all environments on chromosome arm 6DS (psp3200), and the overall phenotypic variance for both trait explained by this marker was above 27%. This locus was also included in determination of KS and PPS in eight environments, suggesting a high value target for wheat improvement. The locus probably exerts its effect on PPS by controlling KS, which results from a combination of SL and FSS. With respect to spike morphology, another consistent MTA were related to gwm484 on chromosome arm 2DS and explained about 35 and 42% of SL and FSS phenotypic variance, respectively. Recently, a stable across environment QTL involved in the determination ear morphology/grain yield were detected on chromosome arm 4AL [50].

## 4. Experimental Section

### 4.1. Plant Material

This study evaluated a collection of 96 winter wheat genotypes (mainly cultivars and advanced breeding lines) which represent germplasm from 21 countries across five continents. They were assembled from a larger core collection created at the Institute of Field and Vegetable Crops, Novi Sad, Serbia on the basis of contrasting expression for 12 traits of agronomic importance such as flowering time, grains per spike, tillering capacity, grain-fill rate and seed size [20]. The collection includes a number of important “founder genotypes” widely used as parents in breeding programs across the world. The detailed list of genotypes and their origins is provided as supplementary information (Electronic Supplementary Table 2).

### 4.2. Field Trials and Experimental Data

Field evaluation was conducted in 2001–2002 (02), 2002–2003 (03) and 2003–2004 (04) at the Centre for Agricultural and Technological Research, situated in the drought-prone Timok region of South-eastern Serbia (43°53′N, 22°17′E, 144 m above sea level). Field plots of the genotypes were subject to one of three treatments: full irrigation (irrigated plots; IP), rain supplied (rainfed plots; RP) and rain-sheltered (*i.e.*, drought-stressed plots; DP). A randomized complete block design with two replicates was used every year of the trial. Seeds were sown in late October in 2001 and 2002, mid-November in 2003. Each plot consisted of 3 rows of 1 m length with plants spaced 20 cm apart. Genotypes within replicates were randomized, but ranked approximately according to height expectations to minimize competition arising between lines growing side-by-side. The seeding rate was approximately 450 seeds m^−2^ in all treatments. Standard agronomic practices were used to provide adequate nutrition and keep the plots free of diseases and weeds. A rain shelter [51] was erected above the plots at the end of the winter period when most of the genotypes were at the tillering stage (typically early March) and this remained in place until crop maturity. A major feature ensuring the success of the shelter was the use of a ditch around the edges of the shelter to catch rain and ensure that soil moisture did not encroach into the rain-sheltered plots. Irrigated plots were watered manually when water in the top 75 cm of soil declined to less than 55% of field capacity *i.e.*, 21% of soil moisture (Vertisol soil type). Rainfed plots received no supplemental irrigation other than rainfall. Water availability from emergence to harvest ranged from 54.3 mm (DP in 2002) to 418 mm (IP in 2004) (Table 6). The season 2001–2002 was particularly dry and severe temperatures (≥30 °C) were experienced during grain filling (June). However, the 2002–2003 season had the greatest number of days where temperature exceeded 30 °C (23 days *in toto*) with the inclement weather starting during anthesis (May).

Plants were scored for the following agronomic, physiological and developmental traits: days to heading (DTH), days to flowering (DTF), days between heading and flowering (HF), stem height (SH), peduncle length (PL), peduncle extrusion (PE), productive tillering (PT), spike length (SL), number of fertile spikelets per spike (FSS), number of sterile spikelets per spike (SSS), spikelet density (SD), number of kernels per spike (KS), number of kernels per spikelet (KSL), number of kernels per m^2^ (KN), thousand grain weight (TKW), production per spike (PPS), spike index (SI), above-ground biomass per plant (BPP), harvest index (HI), flag leaf area (LA), flag leaf width (LW), flag leaf chlorophyll content at flowering date (CH1), flag leaf chlorophyll content three weeks after flowering date (CH2) and grain yield (GY). Twenty-four traits were measured in all, but not all the traits were recorded in every experiment (Table 1).

Early vigor was assessed visually at the 6–7 leaf stage (1 to 5, with 5 being the most vigorous). Days to heading was recorded as the number of days after 31 December when spikes were fully emerged from 50% of plants in a plot. Days to flowering (anthers exerted from the spikelets) was recorded as the number of days after 31 December when 50% of the spikes within a given plot were at this stage. Stem height (cm, from the soil surface to the base of spike), spike length (cm, excluding awns), peduncle length (cm, from the last node to the base of spike), peduncle extrusion (cm, from the ligule of flag leaf to the base of spike), chlorophyll content of flag leaves (SPAD units recorded by hand-held chlorophyll meter, Minolta, Japan) and the flag leaf area (cm^2^, leaf width × leaf length × 0.75) were measured on 20 randomly selected main stems at anthesis in every plot. Productive tillering (spike number/row divided by plant number/row) was recorded at maturity. Twenty main tillers were harvested randomly from each plot to measure the number of fertile and sterile spikelets per spike, spikelet density (fertile and sterile spikelets divided by spike length), number of kernels per spike, number of kernels per spikelet and spike index (kernel weight/total spike weight). The remaining plants in each plot were harvested to determine thousand grain weight (g), above-ground biomass per plant (g), harvest index and grain yield (calculated as t·ha^−1^). Number of kernels per m^−2^ was calculated from the number of spikes present in plot and number of kernels per spike.

A drought susceptibility index (DSI) was calculated using the procedure of Fischer and Maurer [52]. This was expressed by the following relationship: DSI = [1 − (Ys1)/(Yp1)]/SI, where Ys1 and Yp1 are the yields of a genotype under stressed (DP) and non-stressed (IP) conditions, respectively. SII is the stress intensity index which was estimated from: [1 − (Ys2)/(Yp2)], where Ys2 and Yp2 represent the mean yield across all genotypes evaluated under stressed (DP) and non-stressed (IP) conditions, respectively. A stress tolerance index (TOL), [53] was expressed by the following relationship: TOL = Yp-Ys, where Yp is the grain yield under irrigation (IP) and Ys is the grain yield under stress (DP).

### 4.3. Genotype Analysis

Thirty-six SSR markers located across all 21 chromosomes of the hexaploid wheat genome (1–3 SSR markers per chromosome) were used to characterize the genetic diversity of 96 wheat genotypes. In total, 46 loci and 366 alleles were detected, with an average of 7.6 alleles per locus [21]. The markers were selected at random from those optimized for use with an ABI 377 DNA sequencer at the John Innes Centre, Norwich, UK. All accessions were treated as pure lines. A small proportion of heterozygosity was observed (0.7%). The following criteria were used to define the working allele; if the two bands had different intensities, then the stronger band was scored; if the two bands had similar intensities, then the more common allele was retained. Most markers mapped to only one position in the genome but seven markers mapped to more than one and are considered as multilocus markers.

Genomic DNA was extracted from seedlings using a Retsch MM300 Mixer Mill (Haan, Germany) and a Qiagen DNeasy 96 Plant Kit (Qiagen, Mississauga, Ontario), employing the protocol provided in the DNeasy 96 Plant Kit Handbook 08/99, and quantified according to the SYBR Green method of Hopwood *et al.* [54]. SSR amplification was carried out according to Röder *et al.* [55]. Reactions were performed in a Peltier thermocycler (MJ research) using amplification cycles according to the melting temperatures of the primers. Output was analyzed using GeneScan software (version 3.1; PE Applied Biosystems, Foster City, CA, USA, 2000) to measure the molecular size of each SSR allele.

### 4.4. Phenotypic Data Analysis

For each agronomic trait, mixed procedure SAS software (version 9.2; SAS Institute, Inc., Cary, NC, USA, 2009) was used to estimate the variance components according to the following model: *y*_ijk_ = μ + *r*_j_(*e*_i_) + *e*_i_ + *g*_k_ +*ge*_ik_ + ɛ_ijk_ where *r*, *e* and *g* represent replication, environment and accession factors. All factors in the model were considered as random. Broad sense heritability (*h*^2^) was estimated from variance components as described by Holland *et al.* [56]. Separate mixed models where accessions were considered as fixed factors were fitted for producing least square means for all subsequent analyses. Genetic correlations (*r_gij_*) and their standard errors were estimated using the method of Holland [57]. For each correlation coefficient 95% and 99% confidence intervals were constructed as *r_gij_* ± *Z*_(0.05 or 0.01)_*σ*_e_ where is *Z*_(0.05)_ is the value of normal Gaussian distribution and *σ*_e_ is the standard error of the correlation. Correlations were considered as significantly different from zero if the confidence interval did not contain zero [56].

A partial least squares (PLS) regression model [58,59] was performed in order to investigate interaction structure between environments, accessions and agronomic traits. This approach is based on an algorithm which simultaneously modeled (through the dimension reduction) data variation contained in dependent accession by environment matrices of grain yield and agronomic trait, respectively. For the purpose of this study, the dependent matrix was double centered to contain only the interaction effects, whereas the independent matrix was standardized. The relationship among the matrices is represented through latent variables which summarize the data pattern variation [59]. The results of PLS model were presented as a triplot which contained the co-ordinates of environments, accessions (grouped according to their model-based sub-population membership) and agronomic traits. The PLS model algorithm is implemented in R software [60], following the procedure described in Vargas *et al*. [59].

### 4.5. Population Structure

The genetic ancestry of 96 wheat accessions was inferred by a Bayesian model-based clustering approach provided in Structure software [61], with the length of the burn-in period of 10,000 steps followed by 100,000 Monte Carlo Markov Chain replicates using the model allowing for admixture and correlated allele frequencies [62]. The choice of the most likely numbers of sub-populations *K* was carried out by comparing log probabilities of data [Pr[*X*|*K*]] [61]. Ten independent runs of Structure software were performed for each hypothetical number of sub-populations ranging from one to ten. In addition, the effect of population structure on the agronomic traits was assessed by multiple regression analysis using R software [60].

### 4.6. Linkage Disequilibrium

The pairwise linkage disequilibrium (LD) among the pairs of SSR loci was analyzed in according to Witt and Buckler [63]. Prior to LD analysis 10% threshold was used to remove rare alleles in order to overcome their negative bias in LD estimation. LD was measured by a square of the correlation of the allele frequencies (*r*^2^) between SSR loci. The statistical significance of *r*^2^ values was determined by rapid permutation test [64].

### 4.7. Association Analysis

Association analysis among the SSR markers alleles and least squares means of 24 agronomic traits in different environments was performed using a general linear model (GLM) option provided in TASSEL v.3.0 software [64], considering the information about the population structure (*i.e.*, Q matrix) of the selected wheat core collection to control false positive associations. Prior to constructing the association analysis each agronomic trait was normalized and 10% of “minor alleles” were removed from SSR molecular data using algorithm implemented in TASSEL software. The *P* value of marker was used to declare whether a SSR marker was associated with any agronomic trait in any environment and *R*^2^ to determine the fraction of the total variance explained by the marker term.

## 5. Conclusion

In summary, we were not able to recognize any marker related to grain yield under drought stress conditions. However, several markers related to developmental and agronomic traits highly correlated with GY under drought were identified such as gwm389 (3BS) for PT, gwm130 (7AS) for PPS and gwm458 (1DL) for HI. Markers gwm99, gwm458 and gwm130 can be referred as the most specific of all used markers as they are related to only one trait (GY, HI and PPS, respectively) in only one treatment (IP, DP and DP, respectively). The harvest index and PPS had highly significant positive correlations with GY under drought. These markers could therefore be used for marked assisted selection for yield under non-stress (gwm99) and drought stress conditions (gwm458 and gwm130). Contrary, markers psp3200 and gwm484 were found to be associated with yield-related traits in one or more treatments, as well as across all treatments. In addition, marker gwm484 was found to be associated with both drought indices, explaining a relatively high proportion (over 25%) of phenotypic variation. Hence, microsatellite markers psp3200 and gwm484 may be useful for improving drought tolerance in wheat. Our study confirmed the role of major locus for phenology (*Ppd* on chromosome 2DS) for drought-adaptive traits but also highlighted novel locus for environmental adaptability (on chromosome 6DS). A larger collection of genotypes giving more reliable estimates of allele frequencies and a much greater marker density, representing the 21 bread wheat chromosomes, would be required to ensure that identified marker loci were tightly linked to the functional genes.

## Supplementary Materials



## Figures and Tables

**Table 1:** Trait means in irrigated (IP), rainfed (RP) and drought (DP) plots (averaged over 3 years).

Trait	Trait Code †	Means ± SEM			DP/IP

		IP	RP	DP	
Days to heading	DTH(3)	138.9 ± 0.40 ^a^	138.4 ± 0.41 ^a^	134.4 ± 0.45 ^b^	96.8
Days to flowering	DTF(3)	144.2 ± 0.54 ^a^	143.5 ± 0.54 ^a^	139.1 ± 0.56 ^b^	96.5
Heading-flowering (days)	HF(3)	5.46 ± 0.95 ^a^	5.31 ± 0.10 ^a,b^	4.96 ± 0.11 ^b^	90.8
Stem height (cm)	SH(3)	71.3 ± 1.32 ^a^	64.4 ± 1.25 ^b^	65.2 ± 1.28 ^a,b^	91.4
Peduncle length (cm)	PL(2)	30.0 ± 0.70 ^a^	28.0 ± 0.72 ^b^	30.1 ± 0.60 ^a^	100.3
Peduncle extrusion (cm)	PE(2)	11.6 ± 0.55 ^a,b^	10.6 ± 0.56 ^b^	13.0 ± 0.46 ^a^	112.1
Prod. tillering (No/plant)	PT(3)	2.45 ± 0.04 ^a^	2.10 ± 0.03 ^b^	1.28 ± 0.02 ^c^	52.2
Spike length (cm)	SL(3)	7.91 ± 0.10 ^a^	7.62 ± 0.10 ^a^	6.99 ± 0.09 ^b^	88.4
Spike density	SD(3)	2.12 ± 0.02 ^a^	2.13 ± 0.03 ^a^	2.15 ± 0.03 ^a^	101.4
Fertile spikelets per spike	FSS(3)	14.9 ± 0.15 ^a^	14.2 ± 0.14 ^b^	12.1 ± 0.10 ^c^	81.2
Sterile spikelets per spike	SSS(3)	1.58 ± 0.09 ^b^	1.77 ± 0.10 ^b^	2.56 ± 0.11 ^a^	162.0
Kernels per spike	KS(3)	38.5 ± 0.54 ^a^	36.5 ± 0.51 ^a^	28.1 ± 0.38 ^b^	73.0
Kernels per spikelets	KSL(3)	2.36 ± 0.04 ^a^	2.32 ± 0.04 ^a^	1.99 ± 0.03 ^b^	81.8
Kernel number (m^−2^)	KN(3)	18801 ± 272 ^a^	15202 ± 238 ^b^	7369 ± 143 ^c^	39.2
1000 kernel weight (g)	TKW(3)	37.1 ± 0.37 ^a^	35.8 ± 0.36 ^a^	33.5 ± 0.33 ^b^	90.3
Biomass per plant (g)	BPP(3)	7.69 ± 0.11 ^a^	6.19 ± 0.09 ^b^	2.90 ± 0.05 ^c^	37.7
Harvest index	HI(3)	0.44 ± 0.01 ^a^	0.43 ± 0.01 ^a^	0.41 ± 0.00 ^b^	93.2
Production per spike (g)	PPS(3)	1.43 ± 0.03 ^a^	1.31 ± 0.02 ^b^	0.94 ± 0.02 ^c^	65.7
Spike index	SI(3)	0.73 ± 0.00 ^a^	0.72 ± 0.00 ^a^	0.72 ± 0.00 ^a^	98.6
Leaf area (cm^−2^)	LA(2)	29.0 ± 0.50 ^a^	–	24.0 ± 0.45 ^b^	82.8
Leaf width (mm)	LW(2)	22.1 ± 0.20 ^a^	–	20.7 ± 0.22 ^b^	93.7
Chlorophyll at flowering ††	CH1(2)	51.0 ± 0.28 ^a^	–	51.7 ± 0.29 ^a^	101.4
Chlorophyll at gr. filling ††	CH2(2)	32.1 ± 0.58 ^a^	–	30.3 ± 0.71 ^b^	94.4
Grain yield (t·ha^−1^)	GY(3)	6.99 ± 0.12 ^a^	5.49 ± 0.11 ^b^	2.48 ± 0.05 ^c^	35.5

Values in the same row followed by the same letter are not significantly different at the 0.05 probability level.

† The number of years at which each trait was assessed is provided in parentheses;

†† measured in SPAD units.

**Table 2:** Trait heritability in irrigated (IP), rainfed (RP) and drought (DP) plots (averaged over years) and overall (averaged over years and treatments).

Trait	IP	RP	DP	Overall
DTH	0.90	0.90	0.85	0.90
DTF	0.88	0.87	0.84	0.88
HF	0.46	0.48	0.26	0.37
SH	0.92	0.86	0.83	0.89
PL	0.80	0.91	0.89	0.87
PE	0.74	0.87	0.82	0.82
PT	0.66	0.69	0.52	0.52
SL	0.93	0.93	0.88	0.91
SD	0.88	0.88	0.89	0.89
FSS	0.83	0.82	0.77	0.78
SSS	0.84	0.87	0.83	0.85
KS	0.79	0.82	0.78	0.79
KSL	0.68	0.73	0.70	0.73
KN	0.61	0.64	0.38	0.48
TKW	0.87	0.84	0.71	0.81
BPP	0.64	0.57	0.39	0.44
HI	0.82	0.80	0.70	0.80
PPS	0.71	0.76	0.77	0.78
SI	0.72	0.55	0.66	0.64
LA	0.78	-	0.76	0.79
LW	0.72	-	0.78	0.77
CH1	0.75	-	0.70	0.71
CH2	0.66	-	0.58	0.57
GY	0.74	0.75	0.47	0.55

**Table 3:** Genetic correlation between grain yield and 23 other traits in irrigated (IP), rainfed (RP) and drought (DP) plots (averaged over years) and overall (averaged over years and treatments).

Trait	IP	RP	DP	Overall
DTH	−0.30 **	−0.28 *	−0.08	−0.25 *
DTF	−0.36 **	−0.35 **	−0.11	−0.31 **
HF	−0.20	−0.25 *	−0.25 *	−0.22 *
SH	0.22 *	0.27 **	0.31 **	0.27 **
PL	0.21 *	0.18	0.12	0.16
PE	0.22 *	0.14	0.07	0.15
PT	0.36 **	0.35 **	0.33 **	0.40 **
SL	−0.11	−0.08	0.11	−0.05
SD	−0.07	−0.11	−0.15	−0.12
FSS	0.02	0.07	0.33 **	0.11
SSS	−0.42 **	−0.41 **	−0.31 **	−0.42 **
KS	0.16	0.23 *	0.29 **	0.21 *
KSL	0.35 **	0.40 **	0.27 **	0.34 **
KN	0.82 **	0.86 **	0.81 **	0.83 **
TKW	0.64 **	0.63 **	0.57 **	0.61 **
BPP	0.75 **	0.82 **	0.86 **	0.79 **
HI	0.51 **	0.52 **	0.51 **	0.54 **
PPS	0.43 **	0.47 **	0.56 **	0.47 **
SI	0.66 **	0.80 **	0.65 **	0.75 **
LA	−0.20	-	−0.40 **	−0.13
LW	−0.04	-	−0.16	0.04
CH1	0.30 **	-	0.34 **	0.30 **
CH2	0.40 **	-	0.31 **	0.39 **
DSI	0.36 **	0.30 **	−0.16	0.25 **
TOL	0.97 **	0.95 **	0.72 **	0.94 **

*, ** Genetic correlation is significantly different from zero at *P* = 0.05 and *P* = 0.01, respectively.

**Table 4:** Relationship between phenotypic traits and population structure computed using the mean values across treatments.

Trait	Phenotype-Population Structure Associations	

	*R*^2^ (%) a	*P*-Value a
DTH	6.0	0.034
DTF	5.7	0.039
HF	0.0	-
SH	57.8	0.000
PL	53.3	0.000
PE	42.9	0.000
PT	3.6	0.094
SL	0.0	-
SD	3.1	0.118
FSS	5.0	0.054
SSS	12.6	0.002
KS	2.4	0.156
KSL	6.0	0.034
KN	3.6	0.095
TKW	1.2	0.253
BPP	25.6	0.000
HI	19.3	0.000
PPS	3.9	0.084
SI	9.1	0.008
LA	6.1	0.032
LW	3.0	0.120
CH1	12.8	0.001
CH2	12.7	0.001
GY	6.4	0.028
DSI	0.0	-
TOL	2.1	0.179

a *R*^2^ and probability values are from the multiple regression analysis.

**Table 5:** Association of SSR markers with agronomic traits and drought indices.

Trait	Marker	Chromosome Arm	*R*^2^^a^,^b^				MTA Consistancy d

			IP	RP	DP	Overall c	
DTH	gwm257	2BS	**0.137**	**0.136**	**0.132**	**0.134**	6
DTH	psp3200	6DS	0.144	**0.181**	**0.192**	**0.165**	6
DTF	gwm257	2BS	**0.129**	**0.143**	0.125	**0.136**	5
DTF	gwm484	2DS	0.197	**0.229**	**0.252**	**0.228**	5
DTF	psp3200	6DS	0.141	**0.172**	**0.181**	**0.175**	6
PT	gwm484	2DS	**0.311**	**0.253**	**0.293**	**0.288**	6
PT	gwm389	3BS	NS	NS	0.144	NS	2
PT	psp3200	6DS	0.139	0.149	0.160	0.161	6
SL	gwm484	2DS	**0.341**	**0.356**	**0.361**	**0.360**	8
SL	psp3200	6DS	**0.271**	**0.257**	**0.288**	**0.273**	9
FSS	gwm484	2DS	**0.422**	**0.420**	**0.401**	**0.414**	7
FSS	psp3200	6DS	**0.330**	**0.318**	0.302	**0.324**	9
SSS	gwm484	2DS	**0.328**	**0.313**	**0.285**	**0.322**	6
SSS	gwm369	3AS	0.208	NS	NS	NS	4
KS	gwm540	5BS	0.253	0.199	NS	0.238	4
KS	psp3200	6DS	0.284	0.252	0.266	0.262	7
KN	psp3088	2AL	0.138	NS	0.127	0.132	4
HI	gwm458	1DL	NS	NS	0.122	NS	2
HI	gwm484	2DS	0.217	0.202	NS	0.202	4
PPS	psp3200	6DS	0.127	0.152	0.167	0.148	7
PPS	gwm130	7AS	NS	NS	0.165	0.153	3
LA	gwm484	2DS	**0.220**	ND	**0.280**	**0.240**	4
LA	psp3200	6DS	**0.232**	ND	**0.257**	**0.250**	4
LW	gwm484	2DS	NS	ND	**0.289**	**0.258**	2
LW	psp3050	7AS	NS	ND	0.138	0.131	2
LW	psp3200	6DS	**0.230**	ND	**0.244**	**0.243**	4
CH2	gwm484	2DS	0.213	ND	**0.291**	0.259	4
GY	gwm99	1AL	0.144	NS	NS	NS	2
GY	gwm484	2DS	0.223	NS	NS	0.198	3
DSI	gwm484	2DS	NA	NA	NA	0.253	2^e^
TOL	gwm257	2BS	NA	NA	NA	0.149	2^e^
TOL	gwm484	2DS	NA	NA	NA	0.228	2^e^

a *R*^2^ indicates the percentage of the total variation explained;

b NS means not significant at the 0.001 level, ND means no data in this treatment, NA means not possible to calculate;

c From combined analysis across treatments;

d No. of environments (treatment/year combination) in which MTA is highly significant;

e No. of years in which MTA is highly significant;

Bold values refer to MTAs that survived Bonferroni correction for multiple testing.

**Table 6:** Precipitation (mm) during the winter period (November–February) and vegetative phase (March–June), including irrigations, and installation dates for the rain-out shelter.

Season	Nov.–Feb.	March–June			Date of Shelter Installation

		Drought Plots	Rainfed Plots	Irrigated Plots a	
2001/02	54.3	0.0	154.3	+180	28 February
2002/03	158.3	5.8	200.6	+50	12 March
2003/04	195.3	3.5	202.9	+20	8 March

a Amount of water added by irrigation.

## References

[1] IPPC (2007). Working Group II: “Impacts, Adaptation and Vulnerability”. Fourth Assessment Report; Intergovernmental Panel on Climate Change.

[2] Reynolds M.P., Mujeeb-Kazi A., Sawkins M. (2005). Prospects of utilizing plant adaptive mechanisms to improve wheat and other crops in drought and salinity prone environments. Ann. Appl. Biol.

[3] Räisänen J., Hansson U., Ullerstig A., Döscher R., Graham L.P., Jones C., Meier H.E.M., Samuelsson P., Willén U. (2004). European climate in the late 21st century: Regional simulations with two driving global models and two forcing scenarios. Clim. Dyn.

[4] Döll P., Flörke M. (2005). Global-scale Estimation of Diffuse Groundwater Recharge. Frankfurt Hydrology Paper 03.

[5] IPCC (2001). Working Group II: “Impacts, Adaptation and Vulnerability”.

[6] Salekdeh G.S., Reynolds M., Bennett J., Boyer J. (2009). Conceptual framework for drought phenotyping during molecular breeding. Trends Plant Sci.

[7] Stich B., Melchinger A.E. (2010). An introduction to association mapping in plants. CAB Rev. Perspect. Agric. Vet. Sci. Nutr. Nat. Resour.

[8] Quarrie S.A., Steed A., Calestani C., Semikhodskii A., Lebreton C., Steele N., Pljevljakusić D., Waterman E., Weyen J. (2005). A high density genetic map of hexaploid wheat (*Triticum aestivum* L.) from the cross Chinese Spring × SQ1 and its use to compare QTLs for grain yield across a range of environments. Theor. Appl. Genet.

[9] Crossa J., Burgueño J., Dreisigacker S., Vargas M., Herrera-Foessel S.A., Lillemo M., Singh R.P., Trethowan R., Warburton M., Franco J. (2007). Association analysis of historical bread wheat germplasm using additive genetic covariance of relatives and population structure. Genetics.

[10] McIntyre C.L., Mathews K.L., Rattey A., Chapman S.C., Drenth J., Ghaderi M., Reynolds M., Shorter R. (2010). Molecular detection of genomic regions associated with grain yield and yield-related components in an elite bread wheat cross evaluated under irrigated and rainfed conditions. Theor. Appl. Genet.

[11] Maccaferri M., Sanguineti M.C., Demontis A., El-Ahmed A., del Moral L.G., Maalouf F., Nachit M., Nserallah N., Ouabbou H., Rhouma S. (2011). Association mapping in durum wheat grown across a broad range of water regimes. J. Exp. Bot.

[12] Breseghello F., Sorrells M.E. (2006). Association analysis as a strategy for improvement of quantitative traits in plants. Crop Sci.

[13] Neumann K., Kobiljski B., Denčić S., Varshney R.K., Börner A. (2011). Genome-wide association mapping: A case study in bread wheat (*Triticum aestivum* L.). Mol. Breed.

[14] Yang D.L., Jing R.L., Chang X.P., Li W. (2007). Identification of quantitative trait loci and environmental interactions for accumulation and remobilization of water-soluble carbohydrates in wheat (*Triticum aestivum* L.) stems. Genetics.

[15] Yang D.L., Jing R.L., Chang X.P., Li W. (2007). Quantitative trait loci mapping for chlorophyll fluorescence and associated traits in wheat (*Triticum aestivum*). J. Integr. Plant Biol.

[16] Rebetzke G.J., Condon A.G., Farquhar G.D., Appels R., Richards R.A. (2008). Quantitative trait loci for carbon isotope discrimination are repeatable across environments and wheat mapping populations. Theor. Appl. Genet.

[17] Rebetzke G.J., van Herwaarden A.F., Jenkins C., Weiss M., Lewis D., Ruuska S., Tabe L., Fettell N.A., Richards R.A. (2008). Quantitative trait loci for soluble stem carbohydrate production in wheat. Aust. J. Agric. Res.

[18] Wang R., Yu Y., Zhao J., Shi Y., Song Y., Wang T., Li Y. (2008). Population structure and linkage disequilibrium of a mini core set of maize inbred lines in China. Theor. Appl. Genet.

[19] Whitt S.R., Buckler E.S. (2003). Using natural allelic diversity to evaluate gene function. Methods Mol. Biol.

[20] Kobiljski B., Quarrie S.A., Denčić S., Kirby J., Ivegeš M. (2002). Genetic diversity of the Novi Sad wheat core collection revealed by microsatellites. Cell Mol. Biol. Lett.

[21] Dodig D., Zorić M., Kobiljski B., Šurlan-Momirović G., Quarrie S.A. (2010). Assessing drought tolerance and regional patterns of genetic diversity among spring and winter bread wheat using simple sequence repeats and phenotypic data. Crop Pasture Sci.

[22] Somers D.J., Banks T., DePauw R., Fox S., Clarke J., Pozniak C., McCartney C. (2007). Genome-wide linkage disequilibrium analysis in bread wheat and durum wheat. Genome.

[23] Chao S., Zhang V., Dubcovsky J., Sorrells M.E. (2007). Evaluation of genetic diversity and genome-wide linkage disequilibrium among US wheat (*Triticum aestivum* L.) germplasm representing different marker classes. Crop Sci.

[24] Prasad B., Babar M.A., Xu X.Y., Bai G.H., Klatt A.R. (2009). Genetic diversity in U.S. hard red winter wheat cultivars as revealed by microsatellite markers. Crop Pasture Sci.

[25] Cooper M., Woodruff D.R., Eisemann R.L., Brennan P.S., DeLacy I.H. (1995). A selection strategy to accommodate genotype-by-environment interaction for grain yield of wheat: Managed-environments for selection among genotypes. Theor. Appl. Genet.

[26] Trethowan R.M., van Ginkel M., Rajaram S. (2002). Progress in breeding for yield and adaptation in global drought affected environments. Crop Sci.

[27] McCartney C.A., Somers D.J., Humphreys D.G., Lukow O., Ames N., Noll J., Cloutier S., McCallum B.D. (2005). Mapping quantitative trait loci controlling agronomic traits in the spring wheat cross RL4452 X ‘AC Domain’. Genome.

[28] Golabadi M., Arzani A., Mirmohammadi Maibody S.A.M., Sayed Tabatabaei B.E., Mohammadi S.A. (2011). Identification of microsatellite markers linked with yield components under drought stress at terminal growth stages in durum wheat. Euphytica.

[29] Remington D.L., Thornsberry J.M., Matsuoka Y., Wilson L.M., Whitt S.R., Doebley J., Kresovich S., Goodman M.M., Buckler E.S. (2001). Structure of linkage disequilibrium and phenotypic associations in the maize genome. Proc. Natl. Acad. Sci. USA.

[30] Abdurakhmonov I.Y., Kohel R.J., Yu J.Z., Pepper A.E., Abdullaev A.A., Kushanov F.N., Salakhutdinov I.B., Buriev Z.T., Saha S., Scheffler B.E. (2008). Molecular diversity and association mapping of fiber quality traits in exotic G. hirstum L. germplasm. Genomics.

[31] Malysheva-Otto L.V., Ganal M.W., Roder M.S. (2006). Analysis of molecular diversity, population structure and linkage disequilibrium in a worldwide survey of cultivated barley germplasm (*Hordeum vulgare* L.). BMC Genet.

[32] Maccaferri M., Sanguineti M.C., Donini P., Tuberosa R. (2005). Population structure and long range linkage disequilibrium in a durum wheat elite collection. Mol. Breed.

[33] Slafer G.A. (2003). Genetic basis of yield as viewed from a crop physiologist’s perspective. Ann. Appl. Biol.

[34] Zhang L.Y., Liu D.C., Guo X.L., Yang W.L., Sun J.Z., Wang D.W., Zhang A.M. (2010). Genomic distribution of quantitative trait loci for yield and yield related traits in common wheat. J. Int. Plant Biol.

[35] Somers D.J., Isaac P., Edwards K. (2004). A high-density microsatellite consensus map for bread wheat (*Triticum aestivum* L). Theor. Appl. Genet.

[36] Kumar N., Kulwal P.L., Balyan H.S., Gupta P.K. (2007). QTL mapping for yield and yield contribution traits in two mapping populations of bread wheat. Mol. Breed.

[37] Hanocq E., Niarquin M., Heumez E., Rousset M., le Gouis J. (2004). Detection and mapping of QTL for earliness components in a bread wheat recombinant inbred lines population. Theor. Appl. Genet.

[38] Worland A.J. (1996). The influence of flowering time genes on environmental adaptability in European wheats. Euphytica.

[39] Guo Z., Song Y., Zhou R., Ren Z., Jia J. (2010). Discovery, evaluation and distribution of haplotypes of the wheat Ppd-D1 gene. New Phytol.

[40] Ruuska S.A., Rebetzke G.J., van Herwaarden A.F., Richards R.A., Fettell N.A., Tabe L., Jenkins C.L.D. (2006). Genotypic variation in water-soluble carbohydrate accumulation in wheat. Funct. Plant Biol.

[41] Ehdaie B., Alloush G.A., Waines J.G. (2008). Genotypic variation in linear rate of grain growth and contribution of stem reserves to grain yield in wheat. Field Crops Res.

[42] Dreccer M.F., van Herwaarden A.F., Chapman S.C. (2009). Grain number and grain weight in wheat lines contrasting for stem water soluble carbohydrate concentration. Field Crops Res.

[43] Bezant J., Laurie D., Pratchett N., Chojecki J., Kearsey M. (1997). Mapping QTL controlling yield and yield components in a spring barley (*Hordeum vulgare* L) cross using marker regression. Mol. Breed.

[44] Marza F., Bai G.H., Carver B.F., Zhou W.C. (2006). Quantitative trait loci for yield and related traits in the wheat population Ning7840 × Clark. Theor. Appl. Genet.

[45] Kirigwi F.M., van Ginkel M., Brown-Guedira G., Gill B.S., Paulsen G.M., Fritz A.K. (2007). Markers associated with a QTL for grain yield in wheat under drought. Mol. Breed.

[46] Quarrie S.A., Pekić-Quarrie S., Radošević R., Rančić D., Dodig D. (2006). Dissecting a wheat QTL for yield present in a range of environments: From the QTL to candidate genes. J. Exp. Bot.

[47] Quarrie S.A., Dodig D., Pekić S., Kirby J., Kobiljski B. (2003). Prospects for marker-assisted selection of improved drought responses in wheat. Bulg. J. Plant Physiol.

[48] Peleg Z., Fahima T., Krugman T., Abbo S., Yakir D., Korol A.B., Saranga Y. (2009). Genomic dissections of drought resistance in durum wheat × wild emmer wheat recombinant inbreed line population. Plant. Cell Environ.

[49] Dashti H., Yazdi-Samadi B., Ghannadha M., Naghavi M.R., Quarrie S. (2007). QTL analysis for drought resistance in wheat using doubled haploid lines. Int. J. Agric. Biol.

[50] Kobiljski B., Denčić S., Kondić-Špika A., Lohwasser U., Börner A. (2009). Locating stable across environment QTL involved in the determination of agronomic characters in wheat. Cereal Res. Commun.

[51] Dodig D., Quarrie S.A., Stanković S., Milijić S., Denčić S. Characterising Wheat Genetic Resources for Responses to Drought Stress [CD-ROM].

[52] Fischer R.A., Maurer R. (1978). Drought resistance in spring wheat cultivars: I. Grain yield responses. Aust. J. Agric. Res.

[53] Rosielle A.A., Hamblin J. (1981). Theoretical aspects of selection for yield in stress and non-stress environments. Crop Sci.

[54] Hopwood A., Oldroyd N., Fellows S., Ward R., Owen S.A., Sullivan K. (1997). Rapid quantification of DNA samples extracted from buccal scrapes prior to DNA profiling. Biotechniques.

[55] Röder M.S., Korzun V., Wendehake K., Plaschke J., Tixier M.H., Leroy P., Ganal M.W. (1998). A microsatellite map of wheat. Genetics.

[56] Holland J.B., Nyquist W.E., Cervantes-Martinez C.T. (2003). Estimating and interpreting heritability for plant breeding: An update. Plant Breed. Rev.

[57] Holland J.B. (2006). Estimating genotypic correlations and their standard errors using multivariate restricted maximum likelihood estimation with SAS Proc MIXED. Crop Sci.

[58] Aastveit A., Martens H. (1986). ANOVA interaction interpreted by partial least squares regression. Biometrics.

[59] Vargas M., Crossa J., Sayre K., Reynolds M., Ramirez M.E., Talbot M. (1998). Interpreting genotype × environment interaction in wheat using partial least squares regression. Crop Sci.

[60] The R Development Core Team (2008). R: A Language and Environment for Statistical Computing.

[61] Pritchard J.K., Stephens M., Donnelly P. (2000). Inference of population structure using multilocus genotype data. Genetics.

[62] Falush D., Stephens M., Prithchard J.K. (2003). Inference of population structure using multilocus genotype data: Linked loci and correlated allele frequencies. Genetics.

[63] Witt S.R., Buckler E.S. (2003). Using natural allelic diversity to evaluate gene function. Methods Mol. Biol.

[64] Bradbury P.J., Zhang Z., Kroon D.E., Casstevens T.M., Ramdoss Y., Buckler E.S. (2007). TASSEL: Software for association mapping of complex traits in diverse samples. Bioinformatics.

