# Supplementary material for: Genetic and Association Mapping Study of Wheat Agronomic Traits Under Contrasting Water Regimes
Source: Int J Mol Sci. 2012 May 18;13(5):6167–88. doi: 10.3390/ijms13056167 (PMC3382799; doi:10.3390/ijms13056167)
Supplement: Supplementary file 1 [file ijms-13-06167-s001.pdf]

Supplementary Materials

Supplementary Table 1. REML variance of components (± standard errors) of 24 wheat agronomic quantitative traits.

**Trait**	$\sigma^2_{rep(E)}$	σ^2_G	σ^2_E	σ^2_{GE}	σ^2_e
DTH	0.05 ±0.03	31.5 ±4.6	22.3 ±11.2	3.01 ±0.17	0.46 ±0.02
DTF	0.08 ±0.04	27.8 ±4.1	29.1 ±14.6	3.12 ±0.17	0.55 ±0.027
HF	0.002 ±0.002	0.90 ±0.15	1.21 ±0.61	0.87 ±0.05	0.14 ±0.007
SH	0.006 ±0.009	341.1 ±50.1	119.3 ±59.8	38.9 ±2.0	1.35 ±0.06
PL	0.010 ±0.012	59.9 ±8.9	26.3 ±16.7	8.0 ±0.5	1.12 ±0.06
PE	0.001 ±0.006	36.3 ±5.4	13.8 ±8.7	7.01 ±0.48	0.92 ±0.05
PT	0.001 ±0.001	1.3 ±0.2	3.2 ±1.6	0.7 ±0.04	0.08 ±0.04
SL	0.006 ±0.005	22.6 ±3.3	2.2 ±1.1	1.6 ±0.1	0.5 ±0.2
SD	0.003 ±0.003	16.0 ±2.0	0.05 ±0.03	1.5 ±0.1	0.3 ±0.1
FSS	0.002 ±0.002	4.0 ±0.59	1.65 ±0.83	0.93 ±0.05	0.19 ±0.01
SSS	0.003 ±0.007	25.8 ±3.8	2.6 ±1.3	3.3 ±0.2	1.1 ±0.05
KS	0.034 ±0.025	53.4 ±7.9	23.5 ±11.8	12.3 ±0.6	1.88 ±0.09
KSL	0.009 ±0.009	14.0 ±2.0	4.0 ±2.0	4.0 ±0.2	0.9 ±0.4
KN	5.47 ±4.29	7783 ±1219	26970 ±13515	5392 ±2856	347 ±17
TKW	0.03 ±0.02	25.5 ±3.8	8.48 ±4.28	5.57 ±0.30	0.59 ±0.03
BPP	0.003 ±0.005	10.3 ±1.6	49.4 ±24.7	7.9 ±0.4	0.78 ±0.04
HI	0.013 ±0.000	30.0 ±20.0	6.0 ±3.0	7.0 ±4.0	0.80 ±0.04
PPS	0.007 ±0.005	9.0 ±1.0	6.0 ±3.0	2.3 ±0.1	0.34 ±0.01
SI	0.00 ±0.00	0.15 ±0.02	0.02 ±0.001	0.04 ±0.003	0.04 ±0.002
LA	0.008 ±0.019	30.2 ±4.6	16.5 ±13.6	6.1 ±0.59	1.84 ±0.13
LW	0.028 ±0.062	60.0 ±9.3	23.0 ±19.0	12 ±1.2	5.7 ±0.4
CH1	0.003 ±0.007	9.48 ±1.50	3.99 ±3.29	3.17 ±0.29	0.66 ±0.04
GY	0.01 ±0.007	15.1 ±2.3	44.3 ±22.2	7.7 ±0.4	0.42 ±0.02

Supplementary Table 2. List of 96 wheat accessions, their origin and membership corresponding to each of the four subpopulation.

Accession	Origin	Membership of Accession Corresponding to Each Subpopulation			
		A	B	C	D
Acciaio	Italy	0.815	0.008	0.043	0.134
Ai-bian	Japan	0.315	0.010	0.672	0.003
Al Kan Tzao	China	0.063	0.071	0.811	0.055
Ana	Croatia	0.314	0.015	0.610	0.061
Avalon	Great Britain	0.003	0.973	0.005	0.019
Bankuty 1205	Hungary	0.051	0.002	0.006	0.941
BCD 1302/83	Madagascar	0.974	0.006	0.003	0.017
Benni multifloret	USA	0.075	0.005	0.623	0.297
Bezostaya 1	Russia	0.985	0.006	0.005	0.004
Brigand	Great Britain	0.003	0.993	0.003	0.002
Cajeme 71	Mexico	0.671	0.013	0.122	0.194
Capelle Desprez	France	0.003	0.990	0.004	0.003
Centurk	USA	0.047	0.007	0.006	0.940
Ching-Chang 6	China	0.036	0.009	0.943	0.012
Cook	Australia	0.978	0.005	0.007	0.010
Donska polupat.	Russia	0.835	0.098	0.062	0.005
Durin	France	0.085	0.910	0.003	0.002
F 4 4687	Romania	0.236	0.046	0.704	0.014
Florida	USA	0.734	0.159	0.009	0.099
Gala	Argentina	0.983	0.004	0.005	0.008
HAYS 2	USA	0.124	0.075	0.774	0.028
Helios	USA	0.188	0.363	0.066	0.383
Highbury	Great Britain	0.012	0.018	0.932	0.038
Hira	India	0.056	0.005	0.729	0.210
Holly E	USA	0.228	0.202	0.035	0.535
Hope	USA	0.454	0.006	0.005	0.535
Inia 66	Mexico	0.437	0.008	0.411	0.144
INTRO 615	USA	0.007	0.026	0.962	0.005
Ivanka	Serbia	0.948	0.035	0.011	0.006
Kite	Australia	0.006	0.270	0.270	0.455
L 1/91	Serbia	0.560	0.006	0.425	0.010
L 1A/91	Serbia	0.619	0.005	0.365	0.011
L-1	Hungary	0.164	0.138	0.694	0.004
Lambriego Inia	Chile	0.003	0.538	0.014	0.444
Lr 10	USA	0.003	0.002	0.002	0.992
Lr 12	USA	0.168	0.004	0.003	0.825
Magnif 41	Argentina	0.458	0.061	0.443	0.038
Mex. 17 bb	Mexico	0.035	0.024	0.710	0.232
Mex. 3	Mexico	0.021	0.098	0.561	0.320
Mexico 120	Australia	0.004	0.003	0.967	0.025

Supplementary Table 2. *Cont.*

Mina	Serbia	0.222	0.764	0.009	0.005
Minister Dwarf	Australia	0.003	0.992	0.003	0.002
Mironovska 808	Ucraine	0.857	0.131	0.003	0.009
Nizija	Serbia	0.868	0.005	0.109	0.018
Norin 10	USA	0.057	0.087	0.852	0.005
Norin 10/Brev 14	Japan	0.224	0.109	0.260	0.407
Nov. Crvena	Serbia	0.580	0.004	0.015	0.401
Nova Banatka	Serbia	0.750	0.006	0.003	0.242
NS 22/92	Serbia	0.952	0.033	0.010	0.005
NS 33/90	Serbia	0.866	0.007	0.123	0.004
NS 46/90	Serbia	0.917	0.054	0.018	0.011
NS 55-25	Serbia	0.854	0.003	0.003	0.141
NS 559	Serbia	0.249	0.315	0.433	0.003
NS 602	Serbia	0.109	0.073	0.806	0.012
NS 63-24	Serbia	0.875	0.078	0.024	0.024
NS 66/92	Serbia	0.914	0.073	0.007	0.006
NS 74/95	Serbia	0.447	0.355	0.057	0.141
NS 79/90	Serbia	0.935	0.013	0.026	0.026
Peking 11	China	0.004	0.006	0.006	0.984
Phoenix	USA	0.600	0.379	0.005	0.016
PKB Krupna	Serbia	0.986	0.003	0.004	0.007
Pobeda	Serbia	0.980	0.003	0.004	0.012
Purdue 39120	USA	0.003	0.003	0.002	0.992
Purdue 5392	USA	0.003	0.003	0.003	0.992
Purdue/Loras	USA	0.008	0.273	0.003	0.715
Red Coat	USA	0.006	0.013	0.004	0.978
Renesansa	Serbia	0.833	0.004	0.002	0.161
Rusalka	Bulgaria	0.477	0.022	0.497	0.004
Saitama 27	Japan	0.011	0.005	0.973	0.012
Sava	Serbia	0.897	0.040	0.030	0.033
Semillia Eligulata	USA	0.003	0.287	0.706	0.004
Siete Cerros	Mexico	0.009	0.004	0.976	0.011
Slavija	Serbia	0.949	0.041	0.004	0.005
Sofija	Serbia	0.992	0.003	0.002	0.003
Sonalika	India	0.250	0.173	0.205	0.372
Suwwon 92	India	0.010	0.007	0.476	0.508
Szegedi 768	Hungary	0.006	0.006	0.527	0.461
Tibet Dwarf	Tibet	0.434	0.017	0.546	0.003
Timson	Australia	0.007	0.002	0.984	0.007
TJB 990-15	Great Britain	0.004	0.988	0.004	0.004
Tom Thumb	Tibet	0.003	0.992	0.003	0.002
Triple Dirk B	Australia	0.992	0.002	0.003	0.002
Triple Dirk B cont.	Australia	0.990	0.005	0.003	0.002
Triple Dirk S	Australia	0.009	0.025	0.945	0.022

Supplementary Table 2. *Cont.*

Tr. compactum	Serbia	0.149	0.071	0.770	0.010
Tr. sphaerococcum	USA	0.004	0.441	0.206	0.348
UC 65680	USA	0.254	0.004	0.735	0.007
UPI 301	India	0.006	0.099	0.841	0.053
Vel	USA	0.491	0.008	0.047	0.455
Vireo S	Mexico	0.003	0.005	0.697	0.296
WWMCB 2	USA	0.006	0.008	0.983	0.003
ZG 1011	Croatia	0.002	0.004	0.992	0.002
ZG 987/3	Croatia	0.003	0.004	0.991	0.002
ZGK 238/82	Croatia	0.532	0.003	0.423	0.042
ZGK 3/82	Croatia	0.005	0.006	0.984	0.004
ZGKT 159/82	Croatia	0.006	0.008	0.980	0.006

Supplementary Figure 1. Heatmap of the genetic correlations between 24 wheat agronomic traits. The correspondence between colour scale and genetic correlation levels are presented on the right-hand side of the heatmap. Trait codes: BPP = above-ground biomass per plant; CH1 = flag leaf chlorophyll content at flowering date; CH2 = flag leaf chlorophyll content three weeks after flowering date; DTH = days to heading; DTF = days to flowering; FSS = fertile spikelets per spike; GY = grain yield; HF = days between heading and flowering; HI = harvest index; KN = number of kernels per m^2; KS = number of kernels per spike; KSL = number of kernels per spikelet; LA = flag leaf area; LW = flag leaf width; PE = peduncle extrusion; PL = peduncle length; PPS = production per spike; PT = productive tillering; SD = spike density; SH = stem height; SI = spike index; SL = spike length; SSS = sterile spikelets per spike; TKW = one thousand grain weight.

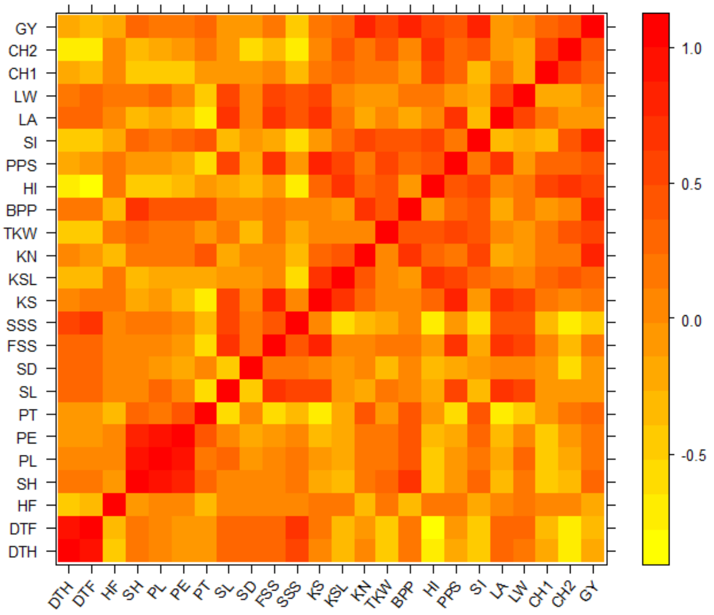

© 2012 by the authors; licensee MDPI, Basel, Switzerland. This article is an open access article distributed under the terms and conditions of the Creative Commons Attribution license (http://creativecommons.org/licenses/by/3.0/).
